# Microglia-Impaired Phagocytosis Contributes to the Epileptogenesis in a Mouse Model of Dravet Syndrome

**DOI:** 10.3390/ijms252312721

**Published:** 2024-11-27

**Authors:** I-Chun Chen, Shih-Yin Ho, Che-Wen Tsai, En-Li Chen, Horng-Huei Liou

**Affiliations:** 1 Department of Pharmacology and Neurology, College of Medicine, National Taiwan University, Taipei 10051, Taiwan; coniewayne@gmail.com (I.-C.C.); aczw2015@gmail.com (C.-W.T.); emily811223@gmail.com (E.-L.C.); 2 Graduate Institute of Biomedical and Pharmaceutical Science, College of Medicine, Fu Jen Catholic University, New Taipei 24205, Taiwan; hoshihyin@gmail.com; 3 Department of Neurology, Fu Jen Catholic University Hospital, Fu Jen Catholic University, New Taipei 24205, Taiwan

**Keywords:** Dravet syndrome, *Scn1a*, microglia, phagocytosis, patch-clamp

## Abstract

Dravet syndrome (DS) is a genetic disorder caused by a deficit in the Nav1.1 channel, leading to drug-resistant epilepsy. The Nav1.1 channel plays a crucial role in microglial cell activation, and microglia are recognized as key mediators of seizures. In this study, we explored the role of microglia in DS-related epileptogenesis using a knock-in mouse model (*Scn1a^E1099X/+^*) that mimics a subset of DS patients. In these DS mice, we observed a significant downregulation of the Nav1.1 channel in microglia. This channel deficit led microglia to adopt a pro-inflammatory state in their quiescent phase. In the LPS-activated state, microglia predominantly exhibited an intermediate morphology rather than the expected fully activated form. The reduced expression of pro-inflammatory cytokines was detected in microglia following treatment with LPS. Notably, we found a significant decrease in the phagocytic ability of microglia in DS mice. Electrophysiological studies revealed an increased immature synaptic activity in the dentate gyrus in DS mice. The impaired microglial phagocytosis of damaged cells, combined with reduced cytokine secretion, may result in an excess of immature synaptic connections, neuronal hyperexcitation, and the formation of abnormal neural circuits in the hippocampus of *Scn1a^E1099X/+^* mice. These changes could potentially contribute to mechanisms relevant to epileptogenesis in DS.

## 1. Introduction

Epilepsy is a brain disorder defined by abnormal electrical discharges in neurons, leading to recurrent seizures. It affects approximately 0.5–1% of the population, with over 60 million individuals impacted globally. Of these, 30% suffer from drug-resistant epilepsy (DRE), which presents significant challenges in treatment [1,2]. The persistence of this condition contributes to major social and economic consequences worldwide. Epileptogenesis refers to the process whereby a previously normal brain undergoes molecular and cellular changes, resulting in the generation of abnormal electrical signals, which ultimately leads to spontaneous recurrent seizures (SRS) [3]. Although the exact mechanisms of epileptogenesis are still unknown, investigating these underlying processes is essential for the development of more effective treatment options for epilepsy patients.

Dravet syndrome (DS) is a severe type of epilepsy where seizures typically emerge within the first year of life, often triggered by increased body temperature from fever or hot baths [4]. Besides hyperthermia-induced seizures (HIS), individuals with DS also suffer from SRS. Patients with DS experience a variety of seizure types, including tonic–clonic, myoclonic, absence, partial, and atonic seizures [5]. Around 80% of the cases are linked to mutations in the *SCN1A* gene, which encodes the Nav1.1 sodium channel [6,7]. While some studies have shown an association between DS severity and *SCN1A* point mutations, particularly those located in the pore region of the sodium channel, these correlations are not consistently found across all research findings [8]. We have developed a knock-in mouse model of the E1099X Nav1.1 truncating mutation (*Scn1a^E1099X/+^*) which carries a mutation analogous to that seen in certain DS patients. These mice begin exhibiting seizures around postnatal day (PD) 20 [9]. Infants with DS are not only prone to HIS but are also at risk for sudden unexpected death, reflecting key features observed in DS patients.

The Nav1.1 channel is predominantly expressed in the soma and axon initial segment (AIS) of parvalbumin-positive (PV^+^) GABAergic neurons [10]. A significant reduction in Nav1.1-expressing GABAergic neurons, along with lower GABA levels in the hippocampus, was observed in *Scn1a^E1099X/+^* mice [9]. Deficiency in Nav1.1 channels leads to decreased action potential firing from the AIS, which in turn reduces GABA release through exocytosis at the axon terminals. This diminished inhibitory signaling to dentate gyrus (DG) granule cells is worsened by an increased release probability at excitatory glutamatergic terminals, further exacerbating the already hyperactive network in DS. Research from our laboratory and others support the idea that insufficient GABA release is a key mechanism in DS epileptogenesis, contributing to brain hyperexcitability [9,10,11]. Since *SCN1A* mutations impair Nav1.1 sodium channels, which are critical for neuronal signaling, particularly in inhibitory interneurons, sodium channel blockers—a common type of anti-epileptic drugs—are generally ineffective for DS and should be avoided. Medications such as carbamazepine and phenytoin can exacerbate symptoms by further disrupting channel function, increasing neuronal excitability and seizure frequency rather than controlling them [12]. While we have shown that an AMPA glutamate receptor inhibitor [13] and an equilibrative nucleoside transporter 1 inhibitor [14] can effectively control HIS in DS mice, treatment options for DS patients remain limited. Standard anti-epileptic medications often fail to manage the severity and frequency of SRS in DS. This underscores the urgent need to identify new therapeutic targets for DS treatment.

The development of epilepsy is marked by neuroinflammation, structural changes, and molecular shifts that result in higher neuronal excitability and the eventual occurrence of SRS. Microglia, the resident immune cells of the brain, possess dynamic processes that constantly monitor their environment. Their role in clearing cellular debris and releasing cytokines is essential for maintaining neural balance [15]. Microglia can adopt two distinct phenotypes, M1 and M2, which are associated with various neurological conditions. The M1 state is linked to inflammation, while M2 is characterized by anti-inflammatory functions [16]. The shape of microglia changes based on the phenotype they express, with highly branched processes during surveillance, shifting to an amoeboid shape in response to specific triggers, aiding in phagocytosis through intracellular vesicles [16,17]. Microglia are increasingly recognized as key players in seizure activity and contributors to epileptogenesis. Morin-Brureau and colleagues [18] studied the differences in microglial behavior in the sclerotic versus non-sclerotic regions of the hippocampus. Their findings showed that microglia in sclerotic areas exhibited more amoeboid shapes with fewer branches, indicating heightened activation. Additionally, pro-inflammatory cytokine levels were elevated in these microglia. In animal models of kainic acid (KA)-induced status epilepticus (SE), inflammatory cytokines like interleukin-1 beta (IL-1β), interleukin-6 (IL-6), and tumor necrosis factor alpha (TNF-α) were notably increased in microglia following seizures [19]. Although most research has focused on the pro-epileptic functions of microglia, they may also have anti-epileptic roles in temporal lobe epilepsy (TLE). In rodent TLE models, SE significantly increased neurogenesis in the DG. However, the newly generated granule cells exhibited abnormal dendrite and axon growth, along with misplaced soma in the hilus, contributing to the creation of hyperexcitable neural circuits [20]. Luo et al. discovered that microglia showed increased CD68, a lysosomal marker, following SE caused by KA. These CD68-positive microglia removed excessive newborn cells in the DG after SE. When microglial activation was pharmacologically inhibited using minocycline, the engulfment of newborn cells decreased, and these cells often ended up in ectopic locations [21]. Moreover, Matsuda et al. [22] reported that activating Toll-like receptor 9 (TLR9) in microglia after KA-induced seizures reduced abnormal neurogenesis. Through the release of cytokines like IL-1β and IL-6, microglia may influence neurogenesis.

Microglia express the Nav1.1, Nav1.5, and Nav1.6 sodium channels under both normal and disease states [23,24]. These sodium channels have been identified as playing a critical role in the activation of microglia. Following spontaneous seizures induced by electrical stimulation, microglia displayed elevated levels of sodium channel subtypes [25]. Additionally, microglia are involved in neurogenesis, contributing to synapse formation, axonal growth, and pruning [26,27]. They achieve this through direct contact with synapses, phagocytosis, and releasing factors that reorganize neuronal circuits during epileptogenesis [28]. Unrestrained reactive gliosis can also result in hippocampal sclerosis, disrupting the brain’s normal regulatory functions and enhancing the likelihood of seizures [29]. Elevated sodium channel expression has been observed in hippocampal sclerosis tissue in patients with DRE [30]. These findings indicate that microglia help maintain the balance of the dentate circuitry by regulating sodium channels in the epileptic brain.

The DS is a form of refractory epilepsy resulting from a deficiency in the Nav1.1 channel. Microglia also express Nav1.1 channels on their surface, and the activation of these sodium channels is crucial for microglial function. Interestingly, the genetic depletion of microglial cells in a zebrafish model of DS led to an exacerbation of neuronal network hyperactivity [31]. This suggests a significant link between the Nav1.1 channel, microglia, and epilepsy. It raises the intriguing possibility that a defective Nav1.1 channel may change microglial function, contributing to epileptogenesis in DS. In this study, we explored the involvement of microglia and Nav1.1 channel deficiency in the development of epilepsy by using a knock-in DS mouse model.

## 2. Results

### 2.1. Nav1.1 Expression in Microglial Cells

**Figure 1:**
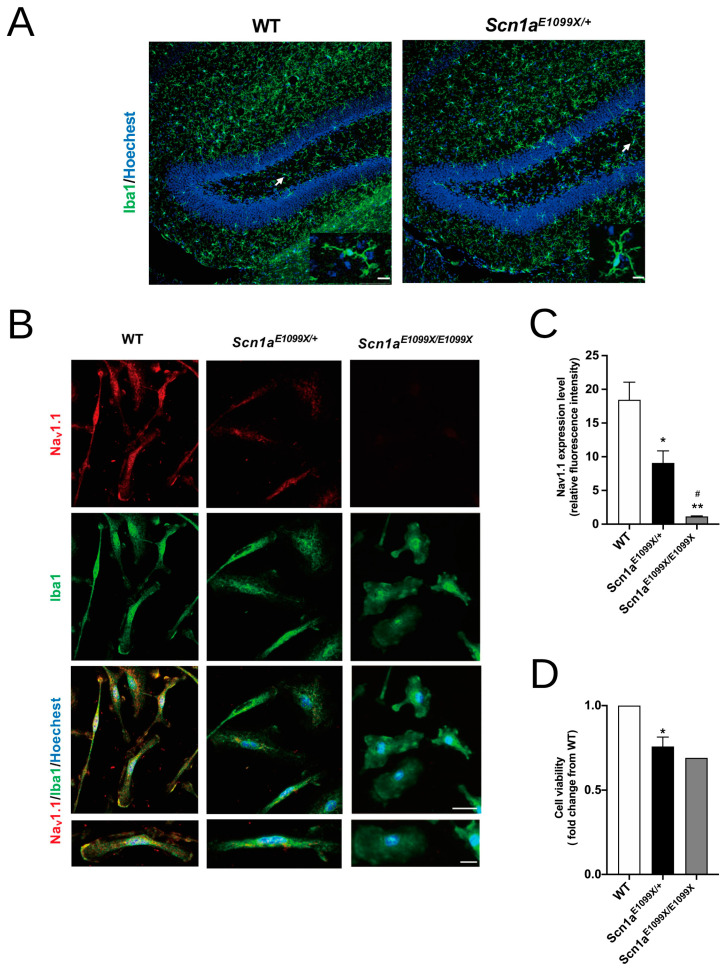
Nav1.1 expression and microglia morphology in the WT, *Scn1a^E1099X/+^*, and *Scn1a^E1099X/E1099X^* mice. (**A**) The immunofluorescence images of the DG region in the WT and *Scn1a^E1099X/+^* mice showing staining for Iba1 (green) to label microglia and hoechst (blue) to label nuclei. The images reveal increased microglial presence and altered morphology in the *Scn1a^E1099X/+^* DG compared to WT. Arrows indicate the resting state of microglial cells in the WT mice, characterized by small soma and long, complex branched processes, and the reactive state of microglial cells in the *Scn1a^E1099X/+^* mice, marked by larger soma and thicker, less branched processes. Scale bars, 10 μm. (**B**) Representative immunofluorescence images showing Nav1.1 (red), Iba1 (green), and hoechst (blue) staining in microglia from the WT, *Scn1a^E1099X/+^*, and *Scn1a^E1099X/E1099X^* mice. Scale bar, 20 μm. Insets show higher magnification of individual microglial cells. Scale bars, 10 μm. (**C**) The quantification of Nav1.1 expression levels in microglia. The Nav1.1 channel expression was significantly reduced in the *Scn1a^E1099X/+^* (N = 3) and *Scn1a^E1099X/E1099X^* (N = 3) mice compared to WT (N = 4). *, # *p* < 0.05; ** *p* < 0.01. (**D**) Cell viability assay from microglia demonstrating decreased cell viability in the *Scn1a^E1099X/+^* (N = 3) and *Scn1a^E1099X/E1099X^* (N = 1) mice compared to WT (N = 4). * *p* < 0.05.

The hippocampus is a primary epileptogenic region [32]. Moreover, electrophysiological recordings in *Scn1a* mutant animals indicate that epileptic activity during HIS may begin in the hippocampus [33]. Thus, we focused on this area to investigate how the *Scn1a* genetic deficit influenced microglial expression. Immunofluorescence images show an increase in microglial presence and morphological changes in the DG of *Scn1a^E1099X/+^* mice compared to WT (Figure 1A). Microglia express voltage-gated sodium channels, particularly TTX-sensitive ones. Next, we evaluated the role of the Nav1.1 channel in microglia using purified primary microglia cultures. Immunocytochemistry results indicated that Nav1.1 channels are present both on the cell membrane and in the cytosol of Iba1-positive microglia in the WT mice. In contrast, Nav1.1 expression was significantly downregulated in *Scn1a^E1099X/+^* and *Scn1a^E1099X/E1099X^* DS mice (Figure 1B). In the *Scn1a^E1099X/+^* mice, the co-expression of the Nav1.1 channels and Iba1-positive microglia was reduced by 50% compared to WT. Additionally, no co-expression of the Nav1.1 channels with Iba1-positive microglia was observed in the *Scn1a^E1099X/E1099X^* mice (Figure 1C), indicating that microglia in these mice do not express Nav1.1. Cell viability, measured via CCK-8 (Cell Counting Kit-8) assays, showed a significant reduction to 60% in microglia from the *Scn1a^E1099X/+^* mice compared to WT (WT, 2.56 ± 0.17, *Scn1a^E1099X/+^* 1.93 ± 0.13, and *Scn1a^E1099X/E1099X^* 1.5; * *p* < 0.05; Figure 1D).

### 2.2. Resting Microglia Morphology and Survival Rate During Nav1.1 Channel Deficiency

**Figure 2:**
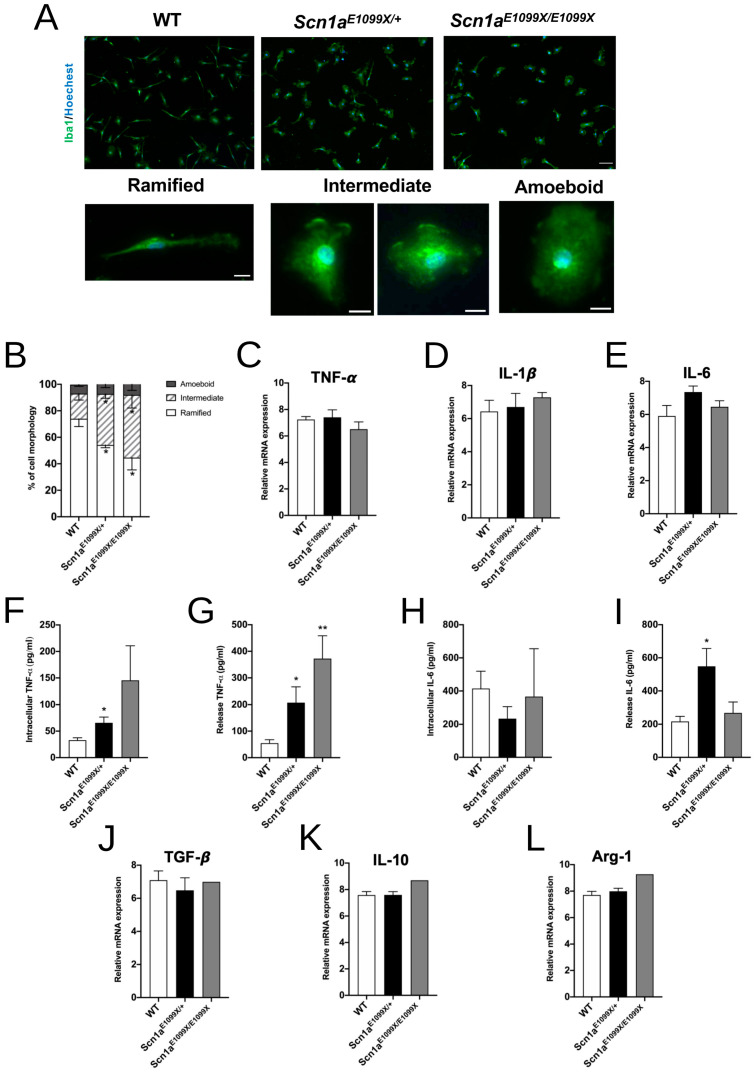
Morphological and cytokine expression analysis of microglia in the WT, *Scn1a^E1099X/+^*, and *Scn1a^E1099X/E1099X^* mice. (**A**) Representative immunofluorescence images showing different morphologies of Iba1^+^ microglia in the DG region of the WT, *Scn1a^E1099X/+^*, and *Scn1a^E1099X/E1099X^* mice. Microglial morphology is categorized as ramified, intermediate, and amoeboid. The ramified microglia display thin and elongated processes, the intermediate form has larger soma and thicker processes, and the amoeboid form shows a large, rounded cell body indicative of an activated state. Scale bars, 20 μm (upper panels) and 5 μm (lower panels). (**B**) The quantification of the percentage of microglia with each morphology type (ramified, intermediate, and amoeboid) in the WT (N = 4), *Scn1a^E1099X/+^* (N = 4), and *Scn1a^E1099X/E1099X^* (N = 3) mice. The *Scn1a^E1099X/+^* and *Scn1a^E1099X/E1099X^* mice show a significant increase in the proportion of intermediate and amoeboid microglia compared to WT, suggesting an enhanced inflammatory state. (**C**–**E**) The relative mRNA expression levels of pro-inflammatory cytokines TNF-α, IL-1β, and IL-6 in microglia from the WT (N = 5), *Scn1a^E1099X/+^* (N = 5), and *Scn1a^E1099X/E1099X^* (N = 4) mice. No significant differences are observed in the mRNA expression levels of these cytokines among the groups. (**F**–**I**) The quantification of intracellular and released levels of pro-inflammatory cytokines. Intracellular TNF-α levels are significantly reduced in the *Scn1a^E1099X/+^* (N = 9) mice compared to WT (N = 8). Released TNF-α levels are significantly elevated in the *Scn1a^E1099X/+^* (N = 12) and *Scn1a^E1099X/E1099X^* (N = 3) mice compared to WT (N = 8). The intracellular IL-6 levels show no significant changes between the groups. The released IL-6 levels are significantly elevated in *Scn1a^E1099X/+^* (N = 11) compared to WT (N = 7), indicating an increase in pro-inflammatory cytokine release. (**J**–**L**) The relative mRNA expression levels of anti-inflammatory markers in the WT (N = 4), *Scn1a^E1099X/+^* (N = 4), and *Scn1a^E1099X/E1099X^* (N = 1) mice. TGF-β, IL-10, and Arg-1 show no significant changes in expression levels across genotypes, indicating that anti-inflammatory signaling may be unaffected at the transcriptional level in *Scn1a^E1099X/+^* microglia. * *p* < 0.05, ** *p* < 0.01.

Microglial cells change shape in response to stimuli and environmental conditions. In their resting state, microglia feature many processes and a small soma, functioning as surveillance cells. However, when external disturbances occur, they shift to an amoeboid shape, characterized by multiple intracellular vesicles, which facilitates phagocytosis. An immunofluorescence analysis was used to assess microglial morphology, classifying cells into resting, intermediate, and activated states (Figure 2A). The morphology distribution in the WT mice showed 74.00 ± 5.90% in the resting state, 19.00 ± 4.92% in the intermediate state, and 6.75 ± 1.32% in the activated state. In heterozygous *Scn1a^E1099X/+^* mice, these percentages shifted to 54.25 ± 2.14% resting, 38.50 ± 3.30% intermediate, and 9.67 ± 4.91% activated. Homozygous *Scn1a^E1099X/E1099X^* mice had 44.67 ± 9.28% in the resting state, 47.33 ± 9.91% in the intermediate state, and 8.33 ± 4.91% activated. These results indicate a significant increase in the intermediate state for microglia in the mutant Nav1.1 mice compared to WT (Figure 2B).

### 2.3. Effect of Scn1a on Pro-Inflammatory and Anti-Inflammatory Cytokines

The release of pro-inflammatory cytokines, including TNF-α, IL-1β, and IL-6, was analyzed using RT-PCR in both the WT and *Scn1a* mutant mice in their quiescent state. The results showed no significant change in the mRNA expression levels of TNF-α (WT, 7.24 ± 0.22; *Scn1a^E1099X/+^*, 7.41 ± 0.56; and *Scn1a^E1099X/E1099X^*, 6.51 ± 0.55), IL-1β (WT, 6.43 ± 0.68; *Scn1a^E1099X/+^*, 6.70 ± 0.82; and *Scn1a^E1099X/E1099X^*, 7.29 ± 0.29), and IL-6 (WT, 5.90 ± 0.63; *Scn1a^E1099X/+^*, 7.35 ± 0.36; and *Scn1a^E1099X/E1099X^*, 6.46 ± 0.37) in the *Scn1a* mutant mice compared to WT (Figure 2C–E). ELISA was conducted to measure both the intracellular and released pro-inflammatory cytokine proteins, including TNF-α and IL-6. Elevated levels of intracellular and released TNF-α and IL-6 proteins were observed in the *Scn1a* mutant mice compared to WT (intracellular TNF-α: WT, 32.98 ± 4.47; *Scn1a^E1099X/+^*, 65.73 ± 10.80; and *Scn1a^E1099X/E1099X^*, 145.4 ± 65.33; released TNF-α: WT, 54.73 ± 13.03; *Scn1a^E1099X/+^*, 207.5 ± 58.45; and *Scn1a^E1099X/E1099X^*, 372.40 ± 86.01; intracellular IL-6: WT, 414.50 ± 104.20; *Scn1a^E1099X/+^*, 234.10 ± 72.53; and *Scn1a^E1099X/E1099X^*, 367.70 ± 287.60; released IL-6: WT, 217.10 ± 30.49; *Scn1a^E1099X/+^*, 548.70 ± 107.8; and *Scn1a^E1099X/E1099X^*, 268.80 ± 65.94, in pg/mL, * *p* < 0.05, ** *p* < 0.01, Figure 2F–I). Furthermore, the mRNA expression of anti-inflammatory cytokines TGF-β (WT, 7.10 ± 0.56; *Scn1a^E1099X/+^*, 6.48 ± 0.76; and *Scn1a^E1099X/E1099X^*, 6.99), IL-10 (WT, 7.59 ± 0.26; *Scn1a^E1099X/+^*, 7.59 ± 0.25; and *Scn1a^E1099X/E1099X^*, 8.7), and Arg1 (WT, 7.71 ± 0.28; *Scn1a^E1099X/+^*, 7.99 ± 0.23; and *Scn1a^E1099X/E1099X^*, 9.27) showed no change in *Scn1a* mutant microglia relative to WT (Figure 2J–L). These results indicate that Nav1.1 deficiency in microglia may promote a pro-inflammatory response even in their quiescent state.

### 2.4. Nav1.1 Impact Microglial Ability to Handle External Impact

**Figure 3:**
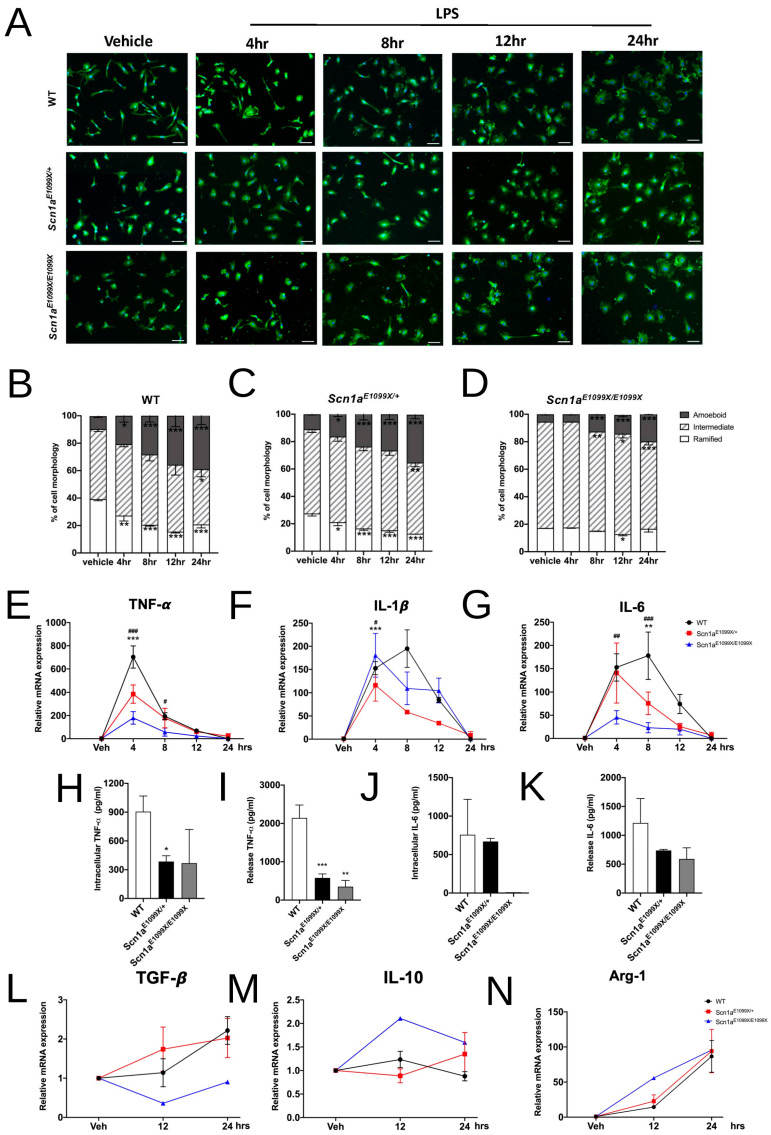
Analysis of microglial morphology and cytokine expression following LPS treatment in the WT and *Scn1a* mutant mice. (**A**) Representative images showing microglial cell morphology in brain sections from the WT and *Scn1a* mutant mice (*Scn1a^E1099X/+^* and *Scn1a^E1099X/E1099X^*) at different time points following LPS treatment (4, 8, 12, and 24 h) compared to vehicle-treated controls. Cells were stained with a microglia-specific marker, and morphology was classified into amoeboid, intermediate, and ramified types. Scale bar, 50 µm. (**B**–**D**) The quantification of microglial morphology in the WT (N = 4), *Scn1a^E1099X/+^* (N = 3), and *Scn1a^E1099X/E1099X^* (N = 2) mice at each time point post-LPS treatment. The bar graphs show the percentage of cells with amoeboid, intermediate, and ramified morphology. Statistical comparisons were made across different time points and genotypes, with significant differences noted. (**E**–**G**) The relative mRNA expression levels of TNF-α, IL-1β, and IL-6 in microglia after LPS stimulation. The expression levels were significantly reduced in the *Scn1a^E1099X/+^* (N = 4), and *Scn1a^E1099X/E1099X^* (N = 4) mice compared to WT (N = 4). (**H**–**K**) The quantification of cytokine levels in the microglia post-LPS treatment. TNF-α and IL-6 in microglia and medium were measured in the WT and *Scn1a* mutant mice. Data represent mean ± SEM. (**L**–**N**) The expression of anti-inflammatory markers in the WT and *Scn1a* mutant mice post-LPS treatment. The graphs show the relative expression levels of TGF-β, IL-10, and Arg-1 at various time points post-LPS injection (WT, N = 4; *Scn1a^E1099X/+^*, N = 1; and *Scn1a^E1099X/E1099X^*, N = 1). Error bars represent mean ± SEM, with statistically significant differences indicated across genotypes and time points. Statistical significance: *, # *p* < 0.05, **, ## *p* < 0.01, and ***, ### *p* < 0.001.

Using lipopolysaccharide (LPS) activation, researchers simulated how microglia respond to external stimuli, such as seizures. Microglial morphology was assessed in the WT, *Scn1a^E1099X/+^*, and *Scn1a^E1099X/E1099X^* mice at various time points: vehicle, 4, 8, 12, and 24 h. By 24 h, the microglial cell numbers had significantly decreased in both the *Scn1a^E1099X/+^* and *Scn1a^E1099X/E1099X^* mice compared to WT (Figure 3A). Microglial morphology was analyzed using Iba1 immunofluorescence, and intermediate morphological changes were tracked over time in all the mice. The WT mice started with 51.00 ± 1.78% in intermediate stages, varying between 52.00 ± 1.87% and 40.25 ± 5.45% over time. The *Scn1a^E1099X/+^* mice began higher at 62.59 ± 3.41%, ending at 51.93 ± 2.93%, while the *Scn1a^E1099X/E1099X^* mice had the highest levels, ranging from 76.93 ± 0.08% to 63.56 ± 2.56%. Activated stages increased over time, with the WT mice rising from 9.50 ± 0.96% at baseline to 39.25 ± 6.69% at 24 h, while the *Scn1a* mutants showed delayed activation, remaining in intermediate stages longer (* *p* < 0.05, ** *p* < 0.01, and *** *p* < 0.001) (Figure 3B–D) (Table 1). This suggests that microglia in the mutant mice remained in an intermediate stage longer, failing to reach the fully activated state seen in the WT mice (* *p* < 0.05, ** *p* < 0.01, and *** *p* < 0.001).

### 2.5. LPS Influence on Pro-Inflammatory and Anti-Inflammatory Function

The relative mRNA expression of microglia under LPS treatment was analyzed using 100 mg/mL LPS and quantitative real-time RT-PCR to evaluate the levels of pro-inflammatory cytokines TNF-α, IL-1β, and IL-6 in the microglia of the WT, *Scn1a^E1099X/+^*, and *Scn1a^E1099X/E1099X^* mice at 4, 8, 12, and 24 h post-treatment. The expression of TNF-α mRNA was significantly reduced in the microglia of the *Scn1a^E1099X/+^* and *Scn1a^E1099X/E1099X^* mice compared to the WT mice at the 4 h (WT, 703.20 ± 95.60; *Scn1a^E1099X/+^*, 385.18 ± 78.62; and *Scn1a^E1099X/E1099X^*, 179.92 ± 54.20; *** *p* < 0.001; ^###^ *p* < 0.001; Figure 3E). However, no significant differences were observed between the mutant and WT mice from 8 to 24 h post-treatment. Similarly, the mRNA levels of IL-1β and IL-6 were significantly lower in the mutant mice compared to WT during the 4 to 8 h after LPS treatment [4 h: IL-1β—WT, 152.59 ± 14.11; *Scn1a^E1099X/+^*, 116.27 ± 34.22; and Scn1a^E1099X/E1099X^, 180.48 ± 47.19; IL-6—WT, 152.70 ± 29.15; *Scn1a^E1099X/+^*, 140.75 ± 64.51; and *Scn1a^E1099X/E1099X^*, 45.59 ± 14.46; 8 h: IL-6—WT, 177.91 ± 50.78; *Scn1a^E1099X/+^*, 75.72 ± 24.29; and *Scn1a^E1099X/E1099X^*, 23.10 ± 11.02; *** *p* < 0.001; ^###^ *p* < 0.001; Figure 3F,G), but this difference was no longer detectable after 12 to 24 h. The protein expression of pro-inflammatory cytokines in microglia from WT, *Scn1a^E1099X/+^*, and *Scn1a^E1099X/E1099X^* mice was analyzed via ELISA following LPS activation. The result showed that the intracellular or released protein levels of TNF-α were significant decreases in *Scn1a* mutant mice compared to WT mice (intracellular TNF-α: WT, 905.50 ± 162.90; *Scn1a^E1099X/+^*, 385.40 ± 60.26; and *Scn1a^E1099X/E1099X^*, 368.70 ± 351.40; released TNF-α: WT, 2143.00 ± 337.40; *Scn1a^E1099X/+^*, 578.30 ± 104.50; and *Scn1a^E1099X/E1099X^*, 349.90 ± 164.20; in pg/mL; * *p* < 0.05, ** *p* < 0.01, and *** *p* < 0.001; Figure 3H,I). No significant differences were found in the intracellular or released protein levels of IL-6 between the Nav1.1 channel mutants and WT mice (Figure 3J,K). For anti-inflammatory cytokine expression, the mRNA levels of TGF-β, IL-10, and Arg1 in microglia were evaluated after IL-4 (20 mg/mL) stimulation in the WT, *Scn1a^E1099X/+^*, and *Scn1a^E1099X/E1099X^* mice at 12 and 24 h. There was no significant difference in the TGF-β, IL-10, and Arg1 mRNA levels between the mutant and WT mice during this time period (TGF-β: WT, 2.22 ± 0. 35; *Scn1a^E1099X/+^*, 2.03 ± 0.50; and *Scn1a^E1099X/E1099X^*, 0.90; IL-10: WT, 0.88 ± 0.10; *Scn1a^E1099X/+^*, 1.35 ± 0.46; and *Scn1a^E1099X/E1099X^*, 1.60; Arg-1: WT, 86.65 ± 22.64; *Scn1a^E1099X/+^*, 94.10 ± 30.88; and *Scn1a^E1099X/E1099X^*, 95.67; Figure 3L–N).

### 2.6. Nav1.1 Affects Microglia Phagocytosis

**Figure 4:**
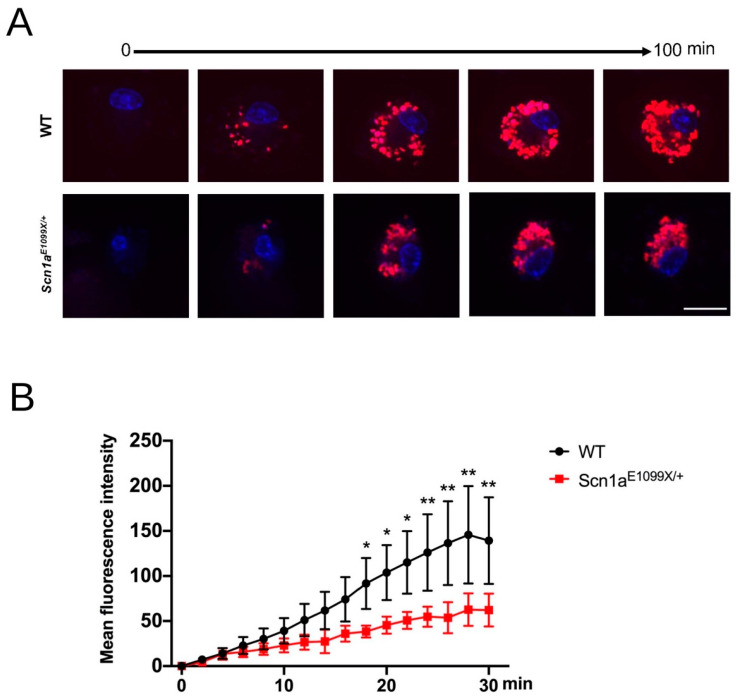
Dynamics of phagocytosis in microglia from the WT and *Scn1a^E1099X/+^* mice. (**A**) Representative fluorescence images showing phagocytosis in microglia isolated from the WT and *Scn1a^E1099X/+^* mutant mice over a time course of 100 min. Cells were incubated with fluorescently labeled particles (red) to monitor phagocytosis, while nuclei were counterstained with a nuclear marker (blue). The WT microglia show a progressive increase in particle uptake over time, whereas the *Scn1a^E1099X/+^* microglia exhibit reduced particle uptake. Scale bars, 10 μm. (**B**) The quantification of the mean fluorescence intensity of phagocytosed particles in the WT and *Scn1a^E1099X/+^* microglia over a 30 min period. The graph shows a time-dependent increase in fluorescence intensity in the WT microglia, indicating efficient phagocytosis, whereas the *Scn1a^E1099X/+^* microglia show significantly reduced fluorescence intensity, reflecting impaired phagocytic activity (WT, N = 3; *Scn1a^E1099X/+^*, N = 3). Data are presented as mean ± SEM, with statistical significance indicated for differences between genotypes at specific time points. Statistical significance: * *p* < 0.05 and ** *p* < 0.01.

Nav1.1 impacts microglia’s ability to perform phagocytosis, a process where cells use their plasma membrane to engulf larger particles, such as pathogens, by forming a phagosome. In the brain, microglia likely contribute to neural circuitry homeostasis by regulating synaptic pruning and formation. To assess microglial phagocytic capacity, we utilized acid-sensitive pHrodo Red *E. coli* bio-particles, which emit a reddish fluorescence in the acidic environment created by phagocytosis. The intensity of this fluorescence correlates with the amount of engulfed particles. We measured this using a laser confocal microscope over 100 min. The results indicated that in the *Scn1a^E1099X/+^* DS mice, the microglial phagocytic ability was reduced by 50% compared to the WT mice (Figure 4A,B). Since one of the key functions of microglia in a non-epileptic brain is synaptic maintenance [34], disruptions in signaling between microglia and neurons could lead to an excess of immature synaptic connections. This is thought to result from the impaired microglial phagocytosis of synapses, further contributing to the neurological dysfunction seen in DS.

### 2.7. Immature Synaptic Activity in the Dentate Gyrus in DS Mice

**Figure 5:**
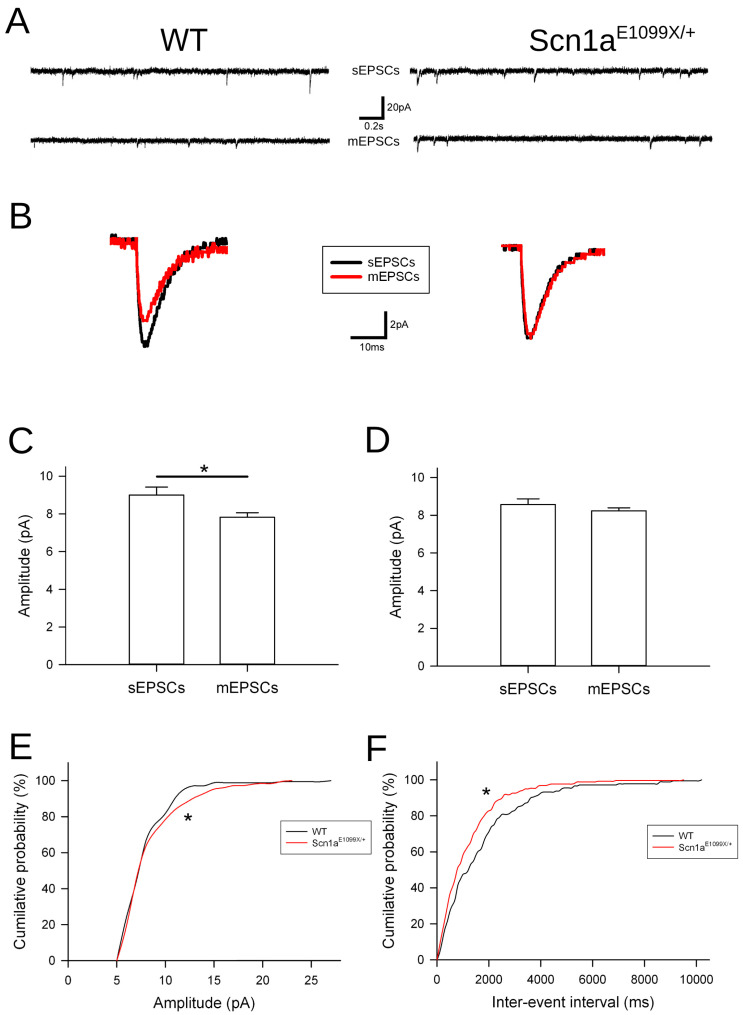
Electrophysiological analysis of synaptic connectivity in the WT and *Scn1a^E1099X/+^* mice. (**A**) The representative traces of sEPSCs and mEPSCs recorded from dentate granule cells in the WT and *Scn1a^E1099X/+^* mice. The recordings display a higher frequency of sEPSCs and mEPSCs in the *Scn1a^E1099X/+^* mice compared to WT, indicating increased synaptic connectivity. Scale bars, 20 pA and 0.2 s. (**B**) Overlapped sEPSC and mEPSC average traces for the WT and *Scn1a^E1099X/+^* mice showing the characteristic kinetics of synaptic events. The traces demonstrate similarities in the sEPSC/mEPSC amplitude ratio in the *Scn1a^E1099X/+^* mice, but with an increased ratio in the WT mice. Scale bars, 2 pA and 10 ms. (**C**) The quantification of sEPSC and mEPSC amplitudes in the WT mice showing a significant difference in amplitude between sEPSCs and mEPSCs. Data are presented as mean ± SEM, and statistical significance is indicated. (**D**) The quantification of sEPSC and mEPSC amplitudes in the *Scn1a^E1099X/+^* mice, showing no significant difference in amplitude between sEPSCs and mEPSCs. Data are presented as mean ± SEM. (**E**) The cumulative probability distribution of mEPSC amplitudes in the WT and *Scn1a^E1099X/+^* mice. The distribution shows a rightward shift in the *Scn1a^E1099X/+^* mice compared to WT, indicating an increased proportion of larger amplitude events. (**F**) The cumulative probability distribution of inter-event intervals for mEPSCs in the WT and *Scn1a^E1099X/+^* mice. The shorter inter-event intervals in the *Scn1a^E1099X/+^* mice reflect increased synaptic event frequency, suggesting enhanced synaptic connectivity (WT, N = 8; *Scn1a^E1099X/+^*, N = 9). * *p* < 0.05.

**Figure 6:**
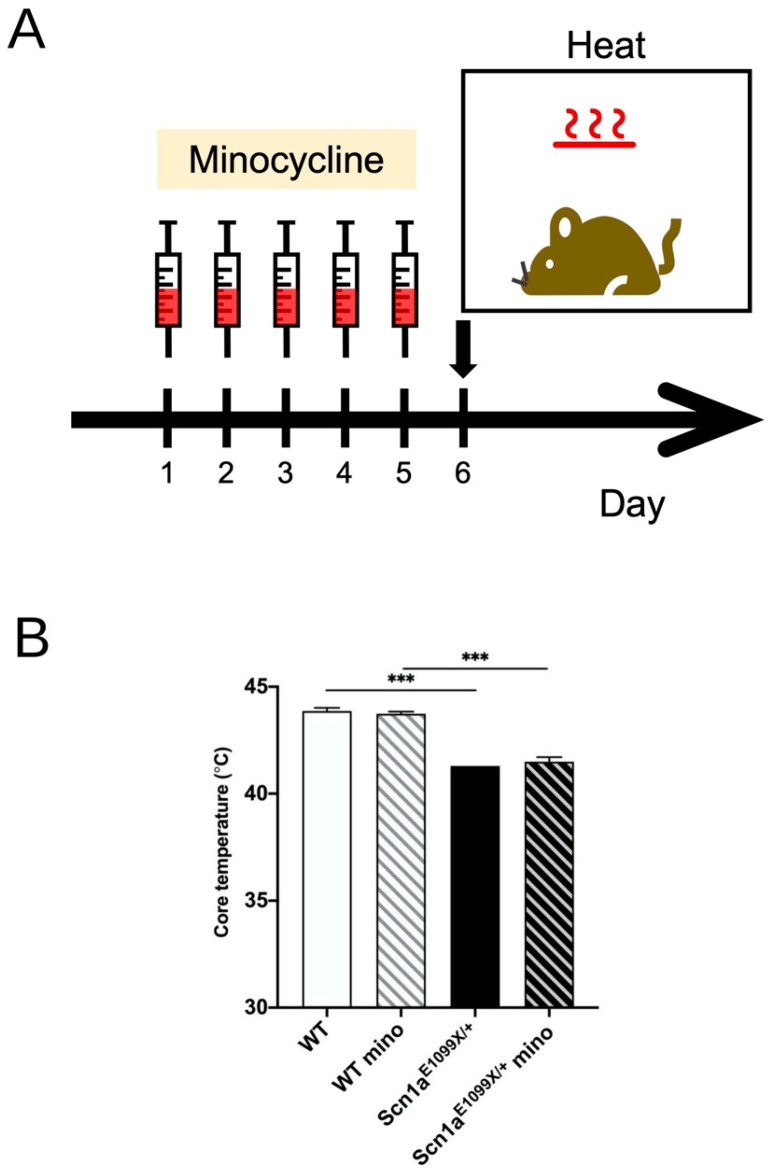
Inhibition of microglia activation by minocycline does not affect the threshold of hyperthermia-induced seizures. (**A**) Experimental scheme for assessing hyperthermia-induced seizures in minocycline-treated mice. (**B**) The quantification of core body temperature in the WT and *Scn1a^E1099X/+^* mice, including those treated with minocycline. The WT mice showed higher core temperatures than the *Scn1a^E1099X/+^* mice. There were no significant effects in the seizure threshold in the WT and *Scn1a^E1099X/+^* mice after minocycline treatment (WT, N = 9; WT mino, N = 8; *Scn1a^E1099X/+^*, N = 3; and *Scn1a^E1099X/+^* mino, N = 3). *** *p* < 0.001.

Microglia play a crucial role in synaptic pruning and refinement, which are essential processes for the maturation of neural circuits [35]. In previous studies conducted by our group, we demonstrated that dendritic spines in the DG granule cells of *Scn1a^E1099X/+^* DS mice failed to undergo the normal synaptic pruning that is observed in the WT mice [9]. To further explore this, we investigated the synaptic connectivity and redundancy of afferent inputs to DG granule cells by utilizing patch-clamp recordings to assess sEPSCs and mEPSCs in both the WT and *Scn1a^E1099X/+^* mice at postnatal week 4. Our findings revealed a marked increase in the frequency of sEPSCs in the *Scn1a^E1099X/+^* mice compared to the WT controls (Figure 5A). While sEPSCs represent both action potential-dependent and spontaneous neurotransmitter release, mEPSCs specifically reflect spontaneous vesicular release. Importantly, the sEPSC/mEPSC amplitude ratio, which typically increases during development and is indicative of the enhanced connectivity and redundancy of afferent inputs [36], was found to be elevated in WT but not in the *Scn1a* mutants (Figure 5B–D). These results suggest immature synaptic connectivity in the *Scn1a^E1099X/+^* DS mice. Additionally, we observed a small yet significant increment in mEPSC amplitude in the *Scn1a* knockout mice (Figure 5E), further supporting the presence of immature synaptic function in these animals. Furthermore, the frequency of mEPSC events was significantly higher in the DS mice compared to WT (Figure 5F), indicating an increased number of synaptic release sites in the *Scn1a* mutant animals. Collectively, these data suggest that microglial dysfunction in the *Scn1a* mutant mice may impair synaptic pruning, leading to excessive and immature synaptic connectivity in the DG granule cells. Next, we examined whether decreased microglia activation by a semisynthetic tetracycline derivative, minocycline, which is known to inhibit microglia activation, would increase susceptibility to hyperthermia-induced seizures in the *Scn1a* mutant mice. To prevent prolonged microglial activation in the *Scn1a^E1099X/+^* mice, we administered minocycline daily intraperitoneal injections to the WT and *Scn1a^E1099X/+^* mice for 5 days (Figure 6A). However, there was no significant effect on the seizure threshold temperature (Figure 6B).

## 3. Discussion

The DS is characterized by a Nav1.1 channel deficiency, leading to the clinical manifestations of SRS. This study revealed a significant downregulation of the Nav1.1 channel and reduced microglial viability in DS mice. Microglia are usually in either a “quiescent” or “activated” state. In the quiescent state, microglia were predominantly (60%) in the intermediate phase in the DS mice. The pro-inflammatory cytokines, such as TNF-α and IL-6, were notably elevated. These findings suggest a pro-inflammatory tendency in microglia lacking the Nav1.1 channel even without external stimulation. Interestingly, in response to LPS or IL-4, the percentage of amoeboid microglia (activated state) in the DS mice was lower than in the WT mice. The pro-inflammatory (TNF-α, IL-1β, and IL-6) but not anti-inflammatory (TGF-β and Arg 1) cytokine expression levels significantly decreased in the stimulated microglia in the DS mice. Notably, microglial phagocytic capacity was markedly reduced, suggesting impaired innate immunity and disrupted homeostasis in the DS brain. Sodium channel activity appears to be crucial for proper microglial function, with reduced activation observed in Nav1.1 channel-deficient microglia following stimulation. The results indicate that impaired microglial ability to phagocytose damaged cells and diminished inflammatory responses may contribute to increased neuronal hyperexcitability and altered neural circuits, potentially aggravating epileptogenesis in DS.

Sodium channel activity regulates various microglial functions [24,37]. In this study, native microglia lacking the Nav1.1 channel shifted toward a pre-amoeboid state, with increased secretion of pro-inflammatory cytokines like IL-1β and TNF-α in the quiescent DS mice. These cytokines exacerbated neuronal hyperactivity and disturbed homeostatic balance, which may have initiated an immune cascade in DS, creating a feedback loop between hyperactive neurons and microglia. This ongoing cycle might promote the development of epileptogenic zones. Another study on DS^A1783V/+^ mice showed microglial morphology shifted to an activated state in the prefrontal cortex and hippocampus without proconvulsant treatment [38]. Similarly, pro-inflammatory cytokine expression, particularly TNF-α, was elevated in the hippocampus. Similar reactive morphologies and increased pro-inflammatory markers were also observed in selectively deleted TSC1 in a microglia mouse model (*TSC1*^Cx3cr1^ CKO mice) [39]. There has been debate regarding whether neuronal overactivity or microglial activation occurs first in epilepsy. It was traditionally believed that neuronal hyperactivity precedes microglial activation in genetically driven epilepsy. However, our findings, along with others, suggest that the genetic deficiency within microglia might be an initial trigger for epileptogenesis in DS. These findings suggest that microglial activation, possibly triggered by Nav1.1 channel defects, could play a role in initiating epileptogenesis, challenging the idea that neuronal hyperactivity precedes microglial activation in genetic epilepsy.

An increase in sodium channel inward current seems to be an early event necessary for microglial activation following spinal cord injury [40]. Applied LPS significantly elevated sodium influx in primary microglia. When sodium channel blockers, such as TTX or phenytoin, were applied, phagocytosis decreased by around 50–60%, and the expression of IL-1α, IL-1β, and TNFα was significantly reduced in LPS-stimulated microglia [24]. Our study found that the phagocytic capacity of microglia in epileptic Nav1.1 channelopathy DS mice was diminished by 50%. The pro-inflammatory cytokine production induced by LPS in activated DS microglia showed marked reductions. Similarly, the conditional knockout mice to delete *TSC1* specifically in microglial cells also demonstrated lower cytokine production in an activated state [39]. However, in the *Scn1Lab*-deficient zebrafish larvae DS model, an M1-like activated amoeboid morphology emerged alongside increased IL-1β expression following recurrent seizures [31]. Our previous research demonstrated decreased excitability in PV-positive interneurons, confirming that Nav1.1 channel dysfunction in DS mice was significant. In DS microglia, we found a 50% reduction in Nav1.1 expression, mirroring the effects of TTX blockade. This may explain the reduced activation of microglia after LPS stimulation in DS mice. Our findings suggest that the innate immune response mediated by Nav1.1-deficient microglia may contribute to the initiation and progression of seizure-induced inflammatory processes in DS.

Microglia-mediated synaptic pruning is essential for facilitating synapse formation and modulating neuronal activity during brain development [41]. In DS mice, we previously demonstrated that the dendritic spines of DG granule cells were not pruned properly [9]. While the exact mechanism of synaptic pruning remains unclear in *Scn1a^E1099X/+^* mice, impaired microglial phagocytosis may have contributed to this synaptic overgrowth before the onset of seizures. This suggests that dysfunction in microglial pruning plays an early role in DS pathology. Our electrophysiological findings revealed that unlike the WT mice, the sEPSC/mEPSC amplitude ratio did not increase in the *Scn1a^E1099X/+^* mice, which usually rises during brain development [36]. Furthermore, we also demonstrated an increased frequency and amplitude of mEPSCs in the DS mice, indicating more excitatory synapses. These immature connections may contribute to the hyperexcitability of DG granule cells, consistent with the findings from other studies. Paolicelli et al. [35] demonstrated that animals lacking CX3CR1 microglial receptors show significant deficits in synaptic pruning, resulting in an excess of dendritic spines and immature synapses. Interestingly, while minocycline reduces microglial activation, it does not increase the vulnerability to hyperthermia-induced seizures in *Scn1a^E1099X/+^* mice. The possible explanation is that suppressing microglia activation alone insufficiently rescues other deficits in these mice, like GABAergic transmission reduction as we previously reported.

A negative correlation was found between microglial phagocytic activity and seizure recurrence. Microglial dysfunction has been reported in patients with DRE who underwent hippocampal resection, as well as in genetic models of epilepsy. In DS, phagocytic impairment likely exacerbates seizure susceptibility and severity. However, enhanced phagocytosis was observed in *TSC1*^Cx3cr1^CKO mice, where elevated mTOR signaling increased microglial ability to clear damaged neurons [39]. Despite previous studies showing mixed results regarding microglial activation in epilepsy, our findings suggest that microglial dysfunction due to Nav1.1 deficiency aggravates seizure severity in DS.

## 4. Materials and Methods

### 4.1. Animals

The wild-type (C57BL/6), heterozygous (*Scn1a^E1099X/+^*), and homozygous (*Scn1a^E1099X/E1099X^*) knock-in mice were obtained from the Animal Center of National Taiwan University College of Medicine (Taipei, Taiwan). All the animal experiments accord with the guidelines of the Institutional Animal Care and Use Committee of the National Taiwan University College of Medicine (IACUC 20190462, 20201018). The mice were kept under a 12 h light–dark cycle, with a controlled temperature of 22 °C ± 2 °C and humidity between 50% and 70%, and had unlimited access to food and water.

### 4.2. Primary Microglia Culture

Primary mixed microglia cultures were prepared from 1- to 3-day-old postnatal mice. Whole brains were dissected in cold dissection buffer (1× HBSS buffer), followed by gentle pipetting with MEM medium and filtering through a 70 μm sterile filter. Cell suspensions were centrifuged at 1000× *g* for 10 min, and the resulting pellet was resuspended in a culture medium (1× Non-essential Amino Acid, 1× sodium pyruvate, 1% penicillin/streptomycin, and 10% FBS in DMEM/F12). The cells were seeded at a density of 2 × 10^6^ in a 25 cm^2^ flask, with medium changes every 3 days. After 14 days, the microglia were harvested by shaking the flasks at 100–120 rpm for 40–60 min at 37 °C, centrifuging the suspension at 1000× *g* for 5 min, and resuspending the cells in the culture medium. The purified microglia were plated in poly-D-lysine-coated 24-well plates and incubated for 1 h at 37 °C. The cells were cultured for one additional day before analysis.

### 4.3. Microglia Morphology

The primary microglial cells were cultured in poly-D-lysine-coated 24-well plates for 1 day. The cells were then fixed using a fixation buffer for 5 min at room temperature and blocked with 3% bovine serum albumin (BSA) for 1 h at room temperature. The microglia were then incubated with a primary antibody against Iba1 (goat anti-Iba1, 1:500, Abcam, Cambridge, UK, Cat # ab5076). Fluorescent images were captured using a Zeiss AxioImager M1 fluorescence microscope (Zeiss, Oberkochen, Germany) and a Zeiss LSM880 confocal laser scanning microscope (Zeiss, Oberkochen, Germany) to analyze the morphology of the microglia. The different morphological stages of the microglia—ramified (with thicker, shorter branches), intermediate (characterized by a larger soma), and amoeboid (with a large, round to variably shaped cell body)—were distinguished based on their structural characteristics. To quantify these morphological differences, the ImageJ software version 1.54a was used to measure the cell area of each type. The results were expressed as the percentage of cells exhibiting ramified, intermediate, or amoeboid morphology following the established methodologies in microglia research [42,43].

### 4.4. Cell Viability Assay

Microglial cell viability was determined using the Cell Counting Kit-8 (CCK-8/WST-8, Sigma-Aldrich, Saint Louis, MO, USA). The cells were seeded at a density of 2 × 10^6^ cells/well in a 96-well plate. After removing the supernatant, the cells were resuspended with WST-8 reagent and incubated for 2 h. Viability was assessed by measuring absorbance at 450 nm using a microplate reader.

### 4.5. Drug Treatment

The microglia were treated with 100 ng/mL lipopolysaccharides (LPSs, Sigma-Aldrich, Saint Louis, MO, USA) for varying durations (4, 8, 12, and 24 h). Additionally, the microglia were incubated with 20 ng/mL IL-4 (Peprotech, Cranbury, NJ, USA) for different time points (12 and 24 h).

### 4.6. Immunocytochemistry

The cells were fixed using a fixation buffer (ethanol: methanol, 1:1) for 5 min at room temperature, followed by three washes with 1× PBS. The cells were blocked with 3% BSA for 1 h at room temperature and incubated overnight at 4 °C with primary antibodies: rabbit anti-Nav1.1 (1:1000, Millipore, Burlington, MA, USA, Cat # AB5204) and goat anti-Iba1 (1:500, Abcam, Cat # ab5076). After the primary antibody incubation, the cells were treated with Alexa Fluor-conjugated secondary antibodies (Alexa 488 or Alexa 546, 1:500, Invitrogen, Waltham, MA, USA) for 1 h at room temperature. The nuclei of the cells were stained with Hoechst 33342 for 30 min at room temperature. Fluorescence images were acquired using a Zeiss AxioImager M1 microscope and a Zeiss LSM880 confocal laser scanning microscope.

### 4.7. RNA Extraction and Quantitative Real-Time PCR

The microglia were collected by centrifuging at 1000× *g* for 5 min, and the total RNA was extracted using the RNeasy mini kit (QIAGEN, Hilden, Germany). cDNA was synthesized with random primers, dNTPs, RNase out, and RT-reaction mix (Promega, Madison, WI, USA). RT-qPCR was performed using the SensiFAST No-ROX One-Step kit (Meridian Bioscience, Cincinnati, OH, USA) containing SYBR Green and predesigned primers. Gene expression levels were normalized to GAPDH.

### 4.8. Phagocytosis Assay

Purified microglial cells were seeded in poly-D-lysine-coated 8-well culture inserts (Ibidi) and allowed to adhere overnight. The pHrodo™ Red *E. coli* Bioparticles^®^ (0.4 mg/mL, Molecular Probe, Eugene, OR, USA) were prepared according to the manufacturer’s protocol for phagocytosis assays. The pHrodo™ Red *E. coli* Bioparticles^®^ fluoresce upon internalization and acidification within the microglia, enabling the visualization and quantification of phagocytosis. To initiate the assay, the culture medium was removed from each well and immediately replaced with 80 μL of the prepared pHrodo™ Red *E. coli* Bioparticles^®^ suspension. Live-cell imaging was performed at 37 °C for 100 min using a Zeiss Laser TIPF/Spinning Disc confocal microscope (Zeiss, Oberkochen, Germany). The total phagocytic activity was quantified by measuring the fluorescence intensity of the microglia using the ImageJ software, allowing for the analysis of the extent of microglial phagocytosis.

### 4.9. Enzyme-Linked Immunosorbent Assay (ELISA)

The microglia were collected by centrifugation at 1000× *g* for 5 min, and the supernatant was retained. The cell pellets were homogenized in 50 mM Tris-HCl (pH 7.4), 150 mM NaCl, 1% Triton X-100, 0.5% sodium deoxycholate, and 0.1% SDS (1× RIPA buffer), and centrifuged at 12,500 rpm for 15 min at 4 °C. Protein detection was performed using a BioLegend ELISA kit (BioLegend, San Diego, CA, USA), and absorbance was measured at 450 nm using a microplate reader.

### 4.10. Electrophysiological Procedures

The electrophysiological experiments in this study were conducted according to previously described methods [14]. Briefly, mouse brains were rapidly removed and placed in a chilled cutting solution (0–4 °C). The cutting solution consisted of the following components (in mM): 110 choline chloride, 0.5 CaCl_2_, 25 glucose, 2 KCl, 1.25 NaH_2_PO_4_, 7 MgSO_4_, 26 NaHCO_3_, 11.6 sodium ascorbate, and 3.1 sodium pyruvate. Coronal brain slices, 300 μm in thickness, were cut using a microslicer (DTK-1000, Dosaka, Kyoto, Japan). After slicing, the brain sections were allowed to recover for at least one hour in artificial cerebrospinal fluid (aCSF) containing (in mM): 125 NaCl, 26 NaHCO_3_, 2 CaCl_2_, 3 KCl, 1 MgCl_2_, 1.25 NaH_2_PO_4_, and 10 glucose, saturated with 95% O_2_ and 5% CO_2_. Whole-cell patch-clamp recordings were obtained from hippocampal dentate granule cells using an Axopatch 200B amplifier (Molecular Devices, San Jose, CA, USA). The neurons were visualized using an infrared-differential interference contrast camera mounted on an upright microscope (BX51WI, Olympus, Tokyo, Japan). Patch electrodes were obtained from borosilicate glass capillaries with a tip resistance of 5–8 MΩ, pulled using a micropipette puller (P97, Sutter Instrument, Novato, CA, USA). The internal pipette solution used for recording was based on potassium gluconate (K-Glu) and contained the following components (in mM): 140 K-Glu, 5 KCl, 10 HEPES, 0.2 EGTA, 2 MgCl_2_, 4 MgATP, 0.3 Na_2_GTP, and 10 Na_2_-phosphocreatine (pH adjusted to 7.2 with KOH, 280–290 mOsm). Data were acquired using a Digidata 1440A digitizer and the pClamp 10 software (Molecular Devices). To isolate spontaneous excitatory postsynaptic currents (sEPSCs), the membrane potential of the neurons was voltage-clamped at −70 mV, and a GABA_A_ receptor antagonist, picrotoxin (100 μM), was applied to block inhibitory currents. To isolate miniature excitatory postsynaptic currents (mEPSCs), tetrodotoxin (TTX, 0.5 μM) was included in the perfusion solution to block action potentials. Series resistance was maintained at 15–30 MΩ, and recordings were discarded if the resistance changed by more than 20%. The recorded signals were filtered at 2 Hz and digitized at 10 Hz. Data analysis, including the detection of sEPSCs and mEPSCs, was performed using the MiniAnalysis Program (Synaptosoft, Decatur, GA, USA).

### 4.11. Hyperthermia-Induced Seizures

Hyperthermia-induced seizures were conducted following the protocol from our previous study [14]. In summary, the mice received intraperitoneal minocycline injections (20 mg/kg) once daily for 5 days (as previously described with modifications) [22]. Seizure susceptibility was assessed one day after the final minocycline injection. The mice were placed in a 48 cm × 45 cm × 42 cm heating chamber equipped with a heating system and observation window. The chamber temperature was increased by approximately 0.5 °C every 10 min until a seizure occurred or 47 °C was reached. Core body temperature was monitored using a rectal temperature probe.

### 4.12. Statistical Analysis

All the data were analyzed using Prism 8. The results are expressed as mean ± S.E.M. Statistical significance was determined using one-way ANOVA or unpaired Student’s *t*-tests.

## 5. Conclusions

In conclusion, the microglia lacking the Nav1.1 channel exhibited a pro-inflammatory profile, enhancing neuronal excitability. Their reduced phagocytic ability and diminished immune responses to environmental changes contributed to the accumulation of cellular debris and excessive immature synaptic connections, leading to hyperexcitability in the hippocampus. This dysfunction likely plays a role in the development of aberrant neural circuits and hippocampal sclerosis, ultimately promoting seizure progression in DS. Interestingly, cannabidiol, an FDA-approved drug for DS, has been shown in various in vitro studies to either inhibit microglial activation or enhance microglial phagocytosis [44,45]. However, further studies are needed to confirm the therapeutic effects of cannabidiol on DS through its influence on microglial function. Taken together, maintaining microglial immune functions and phagocytosis could be a promising therapeutic strategy for reducing seizure frequency in DS patients.

## Figures and Tables

**Table 1:** Comparison of microglia morphology changes between WT and *Scn1a* mutant mice after LPS treatment.

**WT**	**Ramified**	**Intermediate**	**Amoeboid**
vehicle	39.25 ± 1.18% (4)	51.00 ± 1.78% (4)	9.50 ± 0.96% (4)
4 h	27.25 ± 3.99% ** (4)	52.00 ± 1.87% (4)	20.75 ± 4.70% * (4)
8 h	20.50 ± 0.96% *** (4)	51.25 ± 4.64% (4)	28.25 ± 4.54% *** (4)
12 h	15.50 ± 0.96% *** (4)	48.75 ± 7.44% (4)	35.75 ± 7.94% *** (4)
24 h	20.75 ± 2.53% *** (4)	40.25 ± 5.45% (4)	39.25 ± 6.69% *** (4)
***Scn1a^E1099X/+^***	**Ramified**	**Intermediate**	**Amoeboid**
vehicle	27.50 ± 1.80% (3)	61.45 ± 2.23% (3)	11.05 ± 0.53% (3)
4 h	21.09 ± 2.46% * (3)	62.59 ± 3.41% (3)	16.64 ± 2.05% * (3)
8 h	16.53 ± 1.27% *** (3)	59.77 ± 2.91% (3)	24.04 ± 4.49% *** (3)
12 h	15.24 ± 1.47% *** (3)	58.23 ± 3.43% (3)	26.52 ± 3.95% *** (3)
24 h	12.74 ± 0.38% *** (3)	51.93 ± 2.93% ** (3)	34.99 ± 3.01% *** (3)
***Scn1a^E1099X/E1099X^***	**Ramified**	**Intermediate**	**Amoeboid**
vehicle	17.19 ± 0.19% (2)	77.32 ± 0.32% (2)	5.50 ± 0.50% (2)
4 h	17.58 ± 0.58% (2)	76.93 ± 0.08% (2)	5.50 ± 0.50% (2)
8 h	15.06 ± 0.06% (2)	72.36 ± 0.36% ** (2)	12.59 ± 0.42% *** (2)
12 h	12.79 ± 1.22% * (2)	73.02 ± 3.02% * (2)	13.70 ± 1.30% *** (2)
24 h	16.65 ± 2.36% (2)	63.56 ± 2.56% *** (2)	20.30 ± 0.71% *** (2)

Ramified, resting state; Intermediate, activated state; Amoeboid, fully activated state. Numbers in brackets indicate the numbers of mice tested. * *p* < 0.05, ** *p* < 0.01, and *** *p* < 0.001.

## Data Availability

The data that support the findings of this study are available from the corresponding author upon reasonable request.
